# Optimal home and hospital laundering of reusable surgical scrubs: Systematic literature review

**DOI:** 10.4102/hsag.v28i0.2097

**Published:** 2023-05-16

**Authors:** Je’nine Horn-Lodewyk, Tanya Wainwright, K-Cee Lessing, Daniel Otto, Jani H. Fourie

**Affiliations:** 1 Department of Clinical Sciences, Faculty of Health and Environmental Sciences, Central University of Technology, Bloemfontein, South Africa

**Keywords:** reusable surgical scrubs, home laundering, hospital laundering, theatre scrubs, laundering method, infection control

## Abstract

**Background:**

Theatre personnel can spread healthcare-associated infections through contaminated surgical scrubs. Decontamination of surgical scrubs through optimal methods is important to minimise transmission of microorganisms from theatre personnel’s clothing to different areas in the hospital and their homes.

**Aim:**

This study aimed to review the literature on the optimal home and hospital laundering methods for the decontamination of reusable surgical scrubs worn by theatre personnel.

**Method:**

A systematic literature review of previous studies on laundering reusable surgical scrubs was performed. A review question was formulated using the patient, intervention, comparison and outcome (PICO) framework. A literature search was performed using ScienceDirect, Web of Science, ProQuest, EBSCOhost and Google Scholar.

**Results:**

A direct link could be established between the cycle length and water temperature. The higher the water temperature, the shorter the washing cycle required. After a load has been washed in low or medium water temperatures, tumble drying and ironing should follow. Despite the water temperature, a disinfectant must be added to the load.

**Conclusion:**

Health professionals and hospital management should be aware of optimal laundering guidelines for hospital and home laundering as part of infection control. Water temperature, time, mechanical action, type of disinfectant and heat are factors influencing the successful removal of bacteria and other pathogens and represent the baseline of this article.

**Contribution:**

Home-laundering of reusable surgical scrubs should follow strict guidelines. When these specific guidelines are applied, the effects of home-laundered scrubs will not negatively impact either the theatre or the home environment.

## Introduction

The World Health Organization (2017) defines infection prevention and control as a practical, evidence-based approach followed to prevent patients and healthcare workers from contracting an infection. Infection prevention and control have a unique role in patient safety, and universal healthcare quality can prevent the spread of harmful pathogens. Traditional textile-made scrubs used in theatre may be contaminated with microorganisms that might lead to infectious diseases (Mitchell, Spencer & Edmiston 2015). The ideal textile to reduce the spread of microorganisms has not been identified (Goyal et al. 2019). Hence, educating healthcare professionals and hospital management on optimal home and hospital laundering methods of scrubs can provide a practical approach to improving infection prevention and control systems (Goyal et al. 2019; Nordstrom, Reynolds & Gerba 2012).

## Background and rationale for the study

Knowledge, education and training are some of the aspects that may influence healthcare professionals’ compliance or noncompliance with infection prevention and control procedures (Alhumaid et al. 2021). Educating healthcare professionals regarding home laundering can improve the infection control system, thus reducing possible cross-contamination (Goyal et al. 2019; Houang & Hurley 1997). A limited amount of literature on healthcare professionals’ knowledge, education and training on optimal laundering methods could be located. Furthermore, a lack of knowledge among theatre staff on home laundering of their scrubs has been reported (Munoz-Price et al. 2013). Healthcare professionals working in an active theatre environment lack information about infection management in the theatre environment (Zebardast et al. 2019).

Neely and Maley (2000) found that staphylococci and enterococci bacteria that are difficult to eradicate, could survive for long periods of time on fabrics used to make clothes for healthcare professionals. Bacteria isolated from infected surgical wounds were cultured from surgical scrubs prior to surgery (Burden et al. 2013; Moylan, Balish & Chan 1975). These findings suggest that surgical scrubs could be a vector for the transmission of bacterial infections.

Some hospitals permit theatre personnel to launder their scrubs at home because of the increased cost of laundering (Nordstrom, Reynolds & Gerba 2012; Patel, Murray-Leonard & Wilson 2006). Infrastructure issues can increase the cost of laundering as no optimal space for a laundering facility might be available (Lopes et al. 2019). The hospital incurs additional costs for transporting the clothes and having the laundering performed by a private company. In an already cost-constrained hospital environment, staff should know how to optimally launder reusable surgical scrubs in the hospital and at home (Lopes et al. 2019). Subsequently, because of infrastructure-related factors and cost reduction, an increasing number of hospital personnel need to wash their surgical scrubs at home (Lopes et al. 2019; Riley, Laird & Williams 2015). There are different reasons why hospital staff can prefer home laundering, including convenience and a limited number of uniforms supplied by the employer (Riley et al. 2015). Depending on the contracts and/or agreements with personnel, the employer provides an allowance for purchasing scrubs and uniforms. Consequently, when laundering is performed in the hospital laundry, misplaced or stolen scrubs may also be a matter of concern for personnel.

The Association of Surgical Technologists (AST) proposed a set of guidelines compiled from the literature for best practices for laundering surgical scrubs (AST 2017). In the guidelines, the AST emphasises that healthcare delivery organisations must develop and approve policies and protocols for laundering scrubs. The AST guidelines do not recommend home laundering as it cannot be properly monitored. Should theatre personnel be required to perform home laundering, the employer should provide them with information on infection control and laundering guidelines. The AST guidelines also provide information on the handling, storing and transporting of theatre scrubs to prevent contamination with microorganisms. Hence, hospitals have set decontamination laundering protocols related to the infection control of scrubs worn by the theatre personnel (AST 2017; Riley et al. 2015). These protocols should focus on scrubs being laundered at the hospital or at a hospital-approved facility and the manner in which the soiled scrubs are transported to and from the washing facility (AST 2017). Some hospitals allow staff to launder their scrubs at home, even though washing practices may vary. Authors of articles published in the literature could not mutually agree on the proper method (temperature, disinfectant and duration) to launder the surgical scrubs (Al-Benna 2010; Fijan et al. 2007; Fogg 1988; Nordstrom et al. 2012; Sehulster 2015; Smith et al. 1987; Tano & Melhus 2014; Wilson et al. 2007; Zins & Howard 2011). The information in some articles is vague as they only refer to whether high- or low-temperature water is needed and not the specific degrees. The information in the articles also does not contribute to fill the knowledge gap of what guidelines personnel should follow to optimally launder their contaminated scrubs at home without contaminating the home environment (Chiereghin et al. 2020; Riley et al. 2015; Vera, Umadhay & Fisher 2016).

The aim of this study was to review the literature on the optimal home and hospital laundering methods for the decontamination of reusable surgical scrubs worn by theatre personnel. The objective was to critically synthesise and summarise the evidence available to determine the optimal home and hospital laundering methods for the decontamination of reusable surgical scrubs.

## Methods

### Research method and design

A systematic literature review was chosen as a research method to identify and retrieve international evidence on the optimal home and hospital laundering methods for the decontamination of reusable surgical scrubs worn by theatre personnel (Munn et al. 2018).

### Research paradigm

The quantitative paradigm relates to a view of the world and the things in it in a way that involves being able to measure, quantify and verify phenomena (Ellis 2014). In this context, the term ‘quantitative’ refers to a worldview that lends itself to quantification, where study’s findings are quantifiable and countable. The quantitative data were obtained (e.g. water temperature) from the literature with the search terms on the selected electronic databases and recorded in the first Microsoft Excel spreadsheet. Details of the specific home and hospital laundering methods were listed in a second Excel spreadsheet. The results of experimental studies on laundering found in the literature were evaluated. Conclusions were drawn from the different home and hospital laundering methods for reusable surgical scrubs to address the review question.

### Ethical considerations

A systematic literature review is seen as a low-risk study because no personal information is used during data collection and only information obtained from the electronic databases is included in the review (Caldwell 2014). The researchers submitted a detailed protocol that was approved by the Health Sciences Research Ethics Committee (HSREC) of the University of the Free State (ethics reference number UFS-HSD2021/0634/2906) on 14 June 2021.

### Data collection procedure

A systematic literature review was used as the research design. Five steps (Academy of Nutrition and Dietetics 2016) were included in the process, namely:

Step 1: Formulate a focused review question
Step 2: Formulate a search strategy
Step 3: Perform a critical appraisal
Step 4: Summarise the evidence by means of:
  4a. data extraction
  4b. data analysis
Step 5: Summarise the findings.

#### Step 1: The review question

The patient, intervention, comparison and outcome (PICO) model is a widely used tool for structuring research questions for systematic literature reviews (Eriksen & Frandsen 2018). The researchers chose PICO for its ability as a tool to provide a comprehensive search to find relevant publications (Methley et al. 2014). The researchers formulated a review question using the following key elements – population/participants/problem (P); interventions or exposures (I); comparisons or control groups (C); and outcomes of interest (O) (McGrath, Brown & Samra 2012). The use of the PICO model for this study is outlined in Table 1. The review question was specified as ‘*What is the optimal home and hospital laundering method for decontamination of reusable surgical scrubs worn by theatre personnel?*’

**TABLE 1:** Detailing the key elements of patient, intervention, comparison and outcome as applied to this study.

PICO format	Application
**P:** Population /participants/problem/patient/programme	Contaminated reusable surgical scrubs
**I:** Interventions or exposures	Different home and hospital laundering methods for decontamination
**C:** Comparisons or control groups	Different home and hospital laundering methods
**O:** Outcomes of interest	Optimal home and hospital laundering method for decontamination of reusable surgical scrubs

PICO, patient, intervention, comparison and outcome.

#### Step 2: The search strategy

**FIGURE 1:**
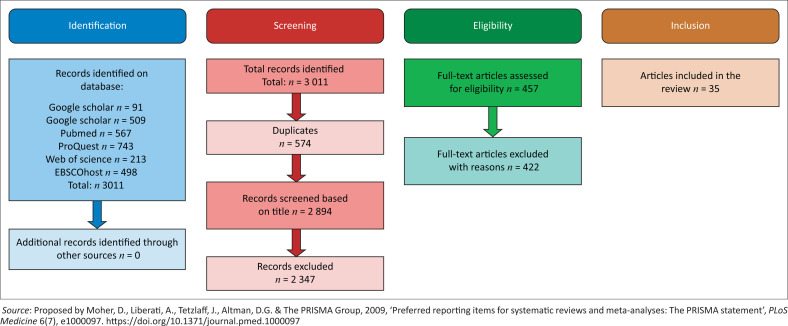
Summary of the literature review search based on the transparent reporting of systematic reviews flow chart.

The researchers retrieved the articles through the use of the following databases available on the Central University of Technology’s (CUT) online library: PubMed, ProQuest, the EBSCOhost platform (selecting the databases Academic Search Premier, Health Sources – Consumer Edition, Health Source: Nursing/Academic Edition, Medline with Full Text) and the Web of Science (WoS) platform (selecting all databases that include Web of Science Core Collection, Derwent Innovations Index, KCI-Korean Journal Database, MEDLINE, Russian Science Citation Index and SciELO Citation Index), Google Scholar and ScienceDirect, as indicated in Figure 1. Based on the findings of a pilot study described here, Boolean operators (‘OR’ and ‘AND’ and ‘NOT’) and wildcards (‘*’) were included in the search. This search strategy initially for the pilot study included the terms ‘surgical scrubs’ OR ‘scrub*’ OR ‘home-launder*’ OR ‘home launder*’ OR ‘launder’ OR ‘wash*’ OR ‘laundering practice*’ OR ‘theatre scrub*’ AND ‘hospital-launder*’ OR ‘hospital launder*’ OR ‘hospital laundry’. Changes needed to be made to the search terms and were improved to ‘surgical scrubs*’ OR ‘theatre scrubs*’ OR ‘surgical attire*’ OR ‘scrub attire*’ AND ‘launder*’ OR ‘home launder*’ OR ‘hospital launder*’ OR ‘washing soiled scrub*’ NOT ‘Money Launder*’.

**Inclusion criteria:** The inclusion criteria for this study were:

- Articles that referred to hospital or home laundering of surgical scrubs
- Primary studies in English
- Research studies such as systematic reviews
- Books
- Published journal articles on primary studies
- Published theses and dissertations
- Conference abstracts
- No restriction was placed on the publication dates of the articles from the search to ensure that significant seminal studies were included.

**Exclusion criteria:** The exclusion criteria for this study were:

- Studies in foreign languages other than English
- Consumer/newspaper articles
- Articles that did not relate to hospital or home laundering of surgical scrubs
- Articles for which only the abstracts were available and full articles could not be accessed or obtained with the assistance of the librarian.

**Reflection from the pilot study:** When conducting a systematic literature review, the researchers should pilot the various steps of the process to increase efficiency and minimise error (Long 2014). The researchers conducted a pilot study with the librarian of the Faculty of Health and Environmental Sciences of the CUT. The authors piloted the search terms by searching them on the databases and the data extraction was also piloted. The initial search terms in the protocol were changed after the pilot study was conducted, but no changes were made to the databases included in the search strategy.

#### Step 3: Perform a critical appraisal

The research process began with three of the researchers (K.L., D.O. and J.H.F.) independently screening the titles and abstracts of the retrieved studies for eligibility against the inclusion criteria. After reading the abstract, the studies that adhered to the inclusion criteria were selected. Following that, the entire articles of the abstracts that were selected as potentially relevant were screened. After the full-text screening, disagreements between the three reviewers were reconciled by the consensus of the fourth and fifth reviewers (J.H.L. and T.W.). To improve rigour, accurate record-keeping was ensured throughout the procedure for verification purposes. The data extracted were transferred into Microsoft Excel 2013 spreadsheets to record and summarise the data collection.

The purpose of a quality assessment is to filter the data and exclude studies that were interpreted with biases. A quality assessment also strengthens the evidence that the researcher wants to use for the article (Taylor, Hussain & Gadoud 2013). The researchers performed a limited quality assessment because of time constraints. For this systematic review, a quality assessment tool (e.g. checklist) was not used. As limited literature was available for this study, all articles were used for data analysis. The researchers evaluated whether the articles met the necessary criteria to be included and if they were valuable and relevant within the specific context of this study; hence, a critical appraisal using more than two reviewers throughout the screening and data collection process reduced reviewer bias (Buscemi et al. 2006). Furthermore, the current evidence was retrieved from the original source and not from a source where it was interpreted from the original. The evidence is mostly from sources published during the past 15 years and every article supplied similar information. These are all characteristics of strong evidence (Berkman et al. 2013).

#### Step 4: Summarise the evidence

*4a. Data extraction:* The researchers extracted the following data from the reviewed studies: authors, article title, journal name, publication year, the year the data were collected and the laundering method. The laundering method included the water washing temperature, washing cycle time, whether disinfectant was used, ironing applied, tumble drying performed and for how long. Furthermore, details of the specific home and hospital laundering methods were fully analysed when included in the articles.

*4b. Data analysis*: The researchers interpreted the data found in the articles through logical and analytical reasoning by applying the process of eliminating irrelevant information. Patterns and relationships between the remaining data, such as the temperature of the water in relation to the washing time, were then identified and presented narratively in Table 1.

#### Step 5: Summarise the findings

The findings were entered into a table to summarise the data and to clearly indicate any relationship between data sets, for example, the higher the water temperature, the shorter the time required for the laundering process. Scrubs laundered at lower water temperatures should be tumble-dried and ironed afterwards.

## Review findings

The Preferred Reporting Items for Systematic Reviews and Meta-Analyses (PRISMA) flow chart summarises the literature review results (Figure 1) (Moher et al. 2009). A total of 3011 articles were identified with the searches. Following the application of the inclusion and exclusion criteria, 35 articles were eligible for full-text analysis. A summary of the articles is provided in Table 2. Thirteen (37.1%) of the 35 articles included for analysis did not provide specific optimal laundering guidelines (see Table 2).

**TABLE 2:** Data extraction of systematic reviews included for the synthesis of evidence.

Reference	Water washing temperature	Duration of washing cycle	Disinfectant	Ironing	Tumble drying	Extra	Hospital and/or home laundering
Sehulster (2015)	60–65.6 °C	Time is inversely proportional to temperature	Chlorine bleach added for both washing temperatures	✓	✓	NI	Hospital
	22–25 °C	NI		✓	✓	NI	Hospital
Halliwell (2012)	40 °C	NI	NI	NI	✓ 30 min	NI	Home
Mitchell et al. (2015)	43 °C	NI	Chlorine bleach added	NI	NI	NI	Hospital and Home
Tano and Melhus (2014)	70 °C	10 min	NI	✓	NI	NI	NI
	< 60 °C	NI	Biocide added	✓	NI	NI	NI
Springer (2008)	60 °C	NI	Bleach added	NI	✓ Highest possible temperature	NI	Hospital and home
Bockmühl et al. (2019)	60 °C	NI	High concentration bleach added	✓	✓	NI	Hospital
Svetanoff et al. (2021)	60 °C	NI	Bleach added	✓	✓	Wash load last and separate	Hospital and home
Wilson et al. (2007)	61 °C	5 min	Bleach added	NI	✓ 85 °C; 5 min	NI	Home
Fijan et al. (2007)	71 °C	3 min	Combination of peroxyacetic acid (7.5%), hydrogen peroxide (20%), and acetic acid (7.5%) for both temperatures and cycles	NI	NI	NI	Hospital
	65 °C	10 min		NI	NI	NI	Hospital
Al-Benna (2010)	40 °C	30–40 min	Bleach added for all washing cycles and temperatures	✓	✓	NI	Home
	65 °C	10 min		✓	✓ 93.3 °C	NI	Home
	65 °C	10 min		✓	✓	NI	Home
	71 °C	3 min		✓	✓ 93.3 °C	NI	Home
	71 °C	3 min		✓	✓	NI	Home
Lakdawala et al. (2011)	65 °C	10 min	NI	NI	✓	NI	Home
Laird and Owen (2020)	60 °C	10 min	NI	NI	NI	NI	Home
Fogg (1988)	71 °C	25 min	Chlorine bleach added	NI	NI	NI	Hospital and home
Fogg (1997)	NI	NI	NI	NI	NI	Does not recommend home laundering.	Hospital
Nordstrom et al. (2012)	71 °C	3 min	Bleach added	NI	NI	NI	Home
Scott et al. (2015)	71 °C	NI	Bleach added	NI	NI	NI	Home
Belkin (1998)	≥ 73.8 °C	NI	Bleach added before rinsing load	NI	✓	Time of washing cycle depends on type of material and the level of contamination.	NI
Hambraeus, Bengtsson and Laurell (1978)	85–90 °C	NI	NI	NI	NI	Maximum pH level of 11 must be maintained throughout the washing cycle.	Hospital
Braswell and Spruce (2012)	NI	NI	NI	NI	NI	Wash the scrubs as a separate load, laundering scrubs as the last load. Wash hands immediately after placing scrubs into the washer.	Hospital
	NI	28 min	NI	✓	NI	NI	Hospital and home
Smith et al. (1987)	Lower water temperature (not specified)	Increased because of lower water temperature	Bleach added	NI	✓ 94°, 25 min	NI	Hospital and home
O’Neale (1997)	NI	NI	NI	N/I	NI	Recommends monitoring of washing cycle for quality control.	Hospital and home
Anonymous (2019)	NI	NI	NI	NI	NI	Recommends monitoring of washing cycle for quality control. Clean scrubs should be covered during transport to hospital.	Hospital
Peterson (2000)	NI	NI	NI	NI	NI	Washing should take place at the hospital and not at other facilities.	Hospital
Petersen (2002)	NI	NI	NI	NI	NI	No reference to specific hospital laundry guidelines. Soiled scrubs not be taken home.	NI
Conner (2000)	NI	NI	NI	NI	NI	No reference to specific hospital laundry guidelines. Soiled scrubs not be taken home.	Home
Zins and Howard (2011)	71–82 °C	30–45 min	Bleach added	NI	NI	NI	Hospital and home
Munoz-Price et al. (2013)	High temperature recommended (not specified)	NI	NI	NI	NI	NI	Home
Leleck et al. (2020)	NI	NI	NI	NI	NI	Method of transporting laundered scrubs determines if scrubs get contaminated before arrival at the hospital.	Home
Spruce (2020)	NI	NI	NI	NI	NI	Home laundering process cannot be monitored (including water temperature and agitation).	Hospital
Opalak and Rivet (2020)	NI	NI	NI	NI	NI	No reference to specific hospital laundry guidelines. Soiled scrubs should not be taken home.	Hospital
Mathias (1998)	Highest temperature achievable by machine.	NI	NI	NI	Immediately after washing with highest possible temperatures achievable.	NI	Home
Anonymous (1998)	NI	NI	NI	NI	NI	No reference to specific hospital laundry guidelines. Soiled scrubs should not be taken home.	Home
Anonymous (1978)	75 °C	NI	NI	NI	NI	Microorganisms can still be found on scrubs laundered at temperatures less than 60 °C.	NI
Jurkovich (1999)	NI	NI	NI	NI	NI	Suggests hospital laundering because personnel do not return scrubs when taken home for laundering.	Home
Anonymous (2016)	NI	NI	NI	NI	NI	No reference to specific hospital laundry guidelines. Soiled scrubs should not be taken home.	Hospital

NI, no information.

The temperature of the water, duration of the washing cycle, type of disinfectant, and whether heat has been applied after laundering, are all factors indicated as affecting an optimal laundering process. Table 2 summarises these factors as concluded from the articles analysed. Twenty-six (74.3%) articles discussed water temperature guidelines for optimal home laundering of reusable surgical scrubs. After analysing the articles included, it was found that 5 (14.3%) articles recommended using a low-water temperature (22 °C to 59 °C) to launder reusable surgical scrubs. A total of 10 (28.5%) articles suggested using medium water temperature (60 °C to 69 °C) and 10 (28.6%) advised using a high-water temperature (70 °C to 90 °C) for optimal laundering of reusable surgical scrubs. User guidelines for domestic washing machines suggest that most washing machines can withstand a maximum water temperature of 60 °C (Repair Aid 2022). Several articles were in favour of hospital laundry facilities because industrial washing machines can withstand higher water temperatures than domestic washing machines used at home. Articles discussing hospital laundering were in agreement regarding water temperatures, time of the washing cycle, and adding a disinfectant. The temperature for hospital laundering was from 70 °C to 90 °C. Articles were unclear regarding tumble drying or ironing after the hospital laundering cycle. It was concluded that the higher the water temperature used for laundering, the shorter the washing time that was required. After scrubs were washed in high temperature water, they were not tumble-dried or ironed. When soiled scrubs were washed with low- or medium-temperature water, they were tumble-dried and ironed afterwards. Regardless of the water temperature, a disinfectant such as bleach was added. No article indicated the concentration of the bleach that should be added to the load.

## Discussion

Although all 35 articles were included in Table 2, 12 of these articles did not include specific hospital or home laundering guidelines. The articles were included because they specifically referred to either hospital laundering or home laundering. In these 12 articles, 5 supported hospital laundering, 4 supported home laundering and 1 supported hospital and home laundering. The remaining two articles did not refer to either hospital or home laundering, neither did they recommend extra information to be considered when laundering soiled scrubs, such as washing your hands after touching the soiled scrubs.

The findings of this study could provide valuable information to healthcare workers and hospitals on optimal home and hospital laundering to improve optimal infection control practices, for example, how the healthcare worker can prevent contaminating their home environment and how the hospital can protect the personnel transporting the soiled scrubs to the laundry facility from bacteria and pathogens. Knowledge of successful decontamination of reusable surgical scrubs is important because bacteria and other pathogens present on the scrubs can spread to the patient, the person wearing the scrubs and to their home environment (Dix 2005).

### Hospital laundering of reusable surgical scrubs

Hospitals should adhere to state regulations regarding the laundering of surgical scrubs as stated in the *Occupational Health and Safety Act* No. *85 of 1993* (OSHA) (South African Government 1993) and the Centers for Disease Control and Prevention (CDC) guidelines (Braswell & Spruce 2012). These regulations include that the hospital should inform the workers handling the soiled scrubs of potential risks, and the water used for the laundry should be disposed of accurately according to prescribed requirements. Another option is that the scrubs should be laundered by an accredited facility (Spruce 2020).

High-temperature water is mainly used for its sanitising effect on the materials. These high temperatures are used in hospital laundering facilities and not during home laundering. Industrial washing machines can withstand these high temperatures and maintain the high temperature throughout the entire washing cycle (Repair Aid 2022).

### Home laundering of reusable surgical scrubs

Without ideal knowledge about laundering methods, other uncontrollable home laundering factors, such as water temperature, agitation and the correct concentration of disinfectants to kill all bacteria and pathogens, need to be taken into consideration (Spruce 2020). Only washing machines should be used and not hand washing. As the washing water could be contaminated, the staff should avoid direct contact with the water and possibly infecting themselves. Evidence of these uncontrollable factors has been reported in a study conducted in 2013, where 11% out of 160 participants did not observe the temperature they used for laundering their reusable surgical scrubs (Munoz-Price et al. 2013). When hospitals allow their staff to launder their scrubs at home to save money, it may create a risk of increased infection rates in the hospital because of the lack of monitoring of home laundering methods (Conner 2000).

The CDC’s 2002 Guidelines for Laundry in Healthcare Facilities stated that surgical scrubs laundered at home had demonstrated no connection with an elevation in infection rates and no microorganisms had been discovered from either home- or hospital-laundered scrubs (Nordstrom et al. 2012). Soiled reusable surgical scrubs should be washed last in a separate load after other laundry has been completed when laundering scrubs at home. Their arms should not be placed in the water of the washing load and their hands should be washed after touching the soiled scrubs (Braswell & Spruce 2012; Springer 2008). The lid of the washing machine should be cleaned thoroughly before the load is removed from the machine and after each wash. The washing machine can be viewed as a site for re-contamination if it is not cleaned properly (Bockmühl, Schages & Rehberg 2019). Hence, the washing machine should be cleaned with bleach (Al-Benna 2010; Fogg 1988; Nordstrom et al. 2012; Scott et al. 2015; Smith et al. 1987; Zins & Howard 2011).

The duration of washing cycles is indirectly proportional to water temperature (Sehulster 2015). Increasing the duration of the washing cycles compensates for low-temperature water (Bockmühl et al. 2019). Microbial decontamination can occur in low-temperature water cycles when factors such as the duration of the cycle are not monitored and controlled throughout the washing cycle (Sehulster 2015). Scrubs washed in water with temperatures below 60 °C will decrease microbial contamination when the correct concentration of disinfectants is added (Springer 2008). No evidence was found that contaminated materials needed to be laundered at water temperatures above 75 °C when disinfectants were used (Fijan et al. 2007). The temperatures achieved when the materials are tumble-dried and ironed compensates for low water temperatures used during washing. Consequently, tumble drying is regarded as compulsory when using low- water temperature (Lakdawala et al. 2011).

Reusable surgical scrubs can be optimally laundered at home under specific conditions. Water temperatures should be between 60 °C and 69 °C for optimal home laundering of reusable surgical scrubs. The reason for this is that domestic washing machines can achieve and maintain medium-water temperature for a washing cycle of at least 10 min. Optimal decontamination occurs at these temperatures (Al-Benna 2010; Fijan et al. 2007). An important consideration with medium temperature is that a disinfectant can also be added, and the laundering process can be followed by tumble drying or ironing the scrubs to ensure that all potential pathogens are destroyed (Al-Benna 2010).

## Limitations of the study

The limitations of the literature review included a restriction on the number of available electronic databases applicable to healthcare. As a result, evidence selection bias caused by missed studies may exist. Older publications found on PubMed were only available in printed format and not electronically online and therefore had to be excluded because they could not be accessed. Therefore, the researchers had to exclude these articles even though they might have contained applicable information. The critical appraisal did not include a tool for quality assessment. However, during the critical appraisal, the researchers scrutinised the methods, results, conclusions and conflict of interests that reduced the risk of bias in the primary studies included.

Decontamination is defined as the level of cleanliness of the surgical scrubs after laundering. This level is determined by whether the bacteria and pathogens, such as staphylococci, are killed (Neely & Maley 2000). Some of the studies included did not provide detail about the decontamination measurement process and rather than stating the killing effect, indicated either reduction or eradication of the microorganism. The studies included had different research questions and study designs. Hence, the methods of measurement of the decontamination were not compared.

## Implications

To ensure optimal hospital and home laundering of theatre scrubs, healthcare professionals and hospital management should be provided with information on the methods by which bacteria and other pathogens are destroyed. If the information on optimal home and hospital laundering methods indicated in this article is correctly applied, it could decrease the microorganism levels present on laundered reusable surgical scrubs and decrease the spread of pathogens. The articles consulted indicated that surgical scrubs should be covered when transported in the hospital, but no transport guidelines were found for home laundering (Anonymous 2019; Leleck et al. 2020). Optimal home and hospital laundering can be simple to implement and provides a practical method to reduce cross-contamination and the transfer of pathogens.

## Recommendations

According to the *Occupational Health and Safety Act No. 85 of 1993*, employees must report any issues to the employer that may result in health and safety incidents (South African Government 1993). Hence, when the hospital requires that theatre personnel must home launder scrubs and they do not have a washing machine, they must report it to the employer. The following simple recommendations were compiled from experimental studies consulted showing the effective killing of microorganisms with specific laundering methods. These recommendations can be applied to home laundering of surgical scrubs:

- Keep contaminated reusable surgical scrubs separate from other clothing.
- Wash reusable surgical scrubs in a separate load.
- Wash reusable surgical scrubs as the last load.
- Immediately wash your hands after placing contaminated reusable surgical scrubs in the washing machine.
- Do not place your hands or arms in the water while the scrubs are washing.
- Wash the scrubs for a minimum of 15 min at water temperatures of 60 °C to 69 °C.
- Monitor the water temperature throughout the washing cycle.
- Chlorine bleach should be added to the wash load. The specific amount is not indicated (Sehulster 2015; Svetanoff et al. 2021).
- Remove the scrubs from the washing machine and clean the washing machine inside, especially the lid.
- Tumble-dry the scrubs for 25 min at the highest possible temperature after washing.
- Iron the scrubs after tumble-drying.

The researchers also compiled simple recommendations from the literature that can be applied for optimal hospital laundering of surgical scrubs:

- Contaminated reusable surgical scrubs should be washed at high temperatures (70 °C to 90 °C) for a minimum of 10 min (Al-Benna 2010; Braswell & Spruce 2012; Fogg 1988; Zins & Howard 2011).
- A combination of peroxyacetic acid (7.5%), hydrogen peroxide (20%) and acetic acid (7.5%) should be added to the wash load to facilitate the eradication of pathogenic bacteria that are difficult to eliminate, such as staphylococci, on the scrubs. The scrubs should be tumble-dried for a minimum of 10 min (Halliwell 2012; Smith et al. 1987).
- The scrubs should be covered and transported back to the hospital without coming into contact with other objects.

## Conclusion

Even though several articles did not recommend home laundering of reusable surgical scrubs, no scientific evidence could be found that this has an influence on the infection rate in healthcare facilities. The guidelines in this review should be followed to successfully remove pathogens from soiled scrubs to ensure good infection control practices. The hospital and home laundering environments of surgical scrubs are different, as well as the equipment that is used. Consequently, recommendations in the guidelines for surgical scrubs laundering at home and in hospitals are varying. These optimal laundering guidelines compiled from the literature include the correct water temperature, time, mechanical action, type of disinfectant and heat. These are all factors that determine whether pathogens are successfully removed from the soiled scrubs. The findings in this review can serve as a basic guideline on recommendations to reduce the transmission of pathogens when home and hospital laundering is performed.
